# Gene Expression Analysis of *Zobellia galactanivorans* during the Degradation of Algal Polysaccharides Reveals both Substrate-Specific and Shared Transcriptome-Wide Responses

**DOI:** 10.3389/fmicb.2017.01808

**Published:** 2017-09-21

**Authors:** François Thomas, Philippe Bordron, Damien Eveillard, Gurvan Michel

**Affiliations:** ^1^Sorbonne Universités, UPMC Univ Paris 06, Centre National de la Recherche Scientifique, UMR 8227, Integrative Biology of Marine Models, Station Biologique de Roscoff Roscoff, France; ^2^Sorbonne Universités, UPMC Univ Paris 06, Centre National de la Recherche Scientifique, FR2424, Analysis and Bioinformatics for Marine Science, Station Biologique de Roscoff Roscoff, France; ^3^Mathomics, Center for Mathematical Modeling, Universidad de Chile Santiago, Chile; ^4^Center for Genome Regulation (Fondap 15090007), Universidad de Chile Santiago, Chile; ^5^Université de Nantes, Laboratoire des Sciences du Numérique de Nantes, Centre National de la Recherche Scientifique, ECN, IMTA Nantes, France

**Keywords:** polysaccharide utilization locus, flavobacteria-algae interactions, regulation of gene expression, operon, microarray

## Abstract

*Flavobacteriia* are recognized as key players in the marine carbon cycle, due to their ability to efficiently degrade algal polysaccharides both in the open ocean and in coastal regions. The chemical complexity of algal polysaccharides, their differences between algal groups and variations through time and space, imply that marine flavobacteria have evolved dedicated degradation mechanisms and regulation of their metabolism during interactions with algae. In the present study, we report the first transcriptome-wide gene expression analysis for an alga-associated flavobacterium during polysaccharide degradation. *Zobellia galactanivorans* Dsij^T^, originally isolated from a red alga, was grown in minimal medium with either glucose (used as a reference monosaccharide) or one selected algal polysaccharide from brown (alginate, laminarin) or red algae (agar, porphyran, ι- or κ-carrageenan) as sole carbon source. Expression profiles were determined using whole-genome microarrays. Integration of genomic knowledge with the automatic building of a co-expression network allowed the experimental validation of operon-like transcription units. Differential expression analysis revealed large transcriptomic shifts depending on the carbon source. Unexpectedly, transcriptomes shared common signatures when growing on chemically divergent polysaccharides from the same algal phylum. Together with the induction of numerous transcription factors, this hints at complex regulation events that fine-tune the cell behavior during interactions with algal biomass in the marine environment. The results further highlight genes and loci that may participate in polysaccharide utilization, notably encoding Carbohydrate Active enZymes (CAZymes) and glycan binding proteins together with a number of proteins of unknown function. This constitutes a set of candidate genes potentially representing new substrate specificities. By providing an unprecedented view of global transcriptomic responses during polysaccharide utilization in an alga-associated model flavobacterium, this study expands the current knowledge on the functional role of flavobacteria in the marine carbon cycle and on their interactions with algae.

## Introduction

Polysaccharides account for a large fraction of the standing stock of organic matter in marine environments. These polysaccharides arise mainly from primary production by phytoplankton in the open ocean (Field et al., 1998), with an added significant contribution of macroalgae in coastal ecosystems (Gattuso et al., 1998). In both micro- and macro-algae, polysaccharides can constitute more than 50% of the dry weight in the form of carbon storage compounds or cell wall constituents, and can be exuded as extracellular material (Kloareg and Quatrano, 1988; Myklestad, 1995; Biddanda and Benner, 1997). Microbial degradation of these diverse and abundant polysaccharide sources is regarded as a crucial bottleneck in the marine carbon cycle, allowing transfer of organic matter to higher trophic levels (Azam and Malfatti, 2007; Gasol et al., 2008; Buchan et al., 2014). Marine algal polysaccharides differ largely from those found in terrestrial plants, with respect to the nature of their monosaccharide units and the presence of sulfated motifs (Popper et al., 2011; De Jesus Raposo et al., 2013; Ficko-Blean et al., 2014). This implies that marine bacteria have evolved dedicated degradation mechanisms to utilize the polysaccharides found in their respective environments. However, knowledge on the bacterial degradation of algal polysaccharides and the regulation of these processes is still scarce in comparison to terrestrial polysaccharides.

Recent studies have shown that marine polysaccharides can be used by diverse specialized taxa, including members of *Bacteroidetes, Gammaproteobacteria, Planctomycetes*, and *Verrucomicrobia* (Martinez-Garcia et al., 2012; Teeling et al., 2012; Lage and Bondoso, 2014; Wietz et al., 2015). In particular, marine *Bacteroidetes* are recognized as key players in the degradation of high molecular weight compounds (Kirchman, 2002; Thomas et al., 2011b). Representatives of the *Flavobacteriia* class are often associated to phytoplankton blooms and macroalgal surfaces and are well known for their ability to utilize a variety of algal polysaccharides (Teeling et al., 2012; Williams et al., 2013; Martin et al., 2014). A decade ago, genome sequencing of the first marine *Flavobacteriia, Gramella forsetii* KT0803^T^ isolated from surface seawater, revealed a substantial suite of carbohydrate active enzymes (CAZymes) such as, 42 glycoside hydrolases (GH), six polysaccharide lyases (PL), eight carbohydrate esterases (CE), and two sulfatases predicted to act on polysaccharides (Bauer et al., 2006). Since then, analysis of several additional genomes from algae-associated flavobacteria uncovered an even greater abundance of CAZyme and sulfatase genes. This includes genomes of bacterial isolates from phytoplankton such as, *Cellulophaga algicola* IC166^T^ (total predicted GH + PL + CE + Sulfatases = 101 genes; Abt et al., 2011) and *Polaribacter* sp. Hel1_85 (104 genes; Xing et al., 2015), as well as isolates from macroalgae such as, *Formosa agariphila* M-2Alg 35-1^T^ (146 genes; Mann et al., 2013) and *Zobellia galactanivorans* Dsij^T^ (236 genes; Barbeyron et al., 2016b). Furthermore, CAZyme-encoding genes are frequently organized in Polysaccharide Utilization Loci (PUL), which are gene clusters typical to *Bacteroidetes* that encode enzymes, binding proteins and transporters required for the breakdown and uptake of complex carbohydrates (Grondin et al., 2017). This genomic knowledge points at a specialization of marine *Flavobacteriia* toward polysaccharide utilization in the environment. However, transcriptomic profiling is required to understand under which conditions these degradation capacities are expressed and the regulations involved, and ultimately deepen our comprehension of algae-flavobacteria interactions. To date, transcriptome-wide gene expression studies on marine *Flavobacteriia* have mainly focused on light-induced responses associated with proteorhodopsin-enhanced growth in members of the genus *Dokdonia* (Kimura et al., 2011; Gómez-Consarnau et al., 2016) and on the response of *Cellulophaga baltica* to viral infection (Howard-Varona et al., 2017). A first transcriptome-wide profiling was reported for the surface seawater isolate *Gramella flava* JLT2011^T^, to characterize its response to the polysaccharides xylan and pectin (Tang et al., 2017). To our knowledge, transcriptome-wide responses linked to polysaccharide degradation have never been investigated in flavobacteria isolates from algae and are still understudied even in other alga-associated bacterial phyla. For instance, Zhu et al. (2016) recently reported a complete gene expression profiling of *Bacillus weihaiensis* Alg07, a *Firmicutes* isolated from rotting seaweed. Using RNA-seq, they showed that *B. weihaiensis* overexpresses several genes involved in alginate metabolism, including genes encoding degradation enzymes, putative transporters, and regulators (Zhu et al., 2016).

In the present study, we explored transcriptome-wide gene expression of *Z. galactanivorans* Dsij^T^ grown on a diverse set of algal polysaccharides. Members of the *Zobellia* genus are marine flavobacteria often found on the surface of healthy green, red, and brown macroalgae (Hollants et al., 2013; Martin et al., 2015; Marzinelli et al., 2015). Among the five currently described *Zobellia* species, *Z. galactanivorans* Dsij^T^ has become a model organism for polysaccharide degradation by marine flavobacteria. Originally isolated from the red alga *Delesseria sanguinea* (Hudson) J. V. Lamouroux 1813 (Barbeyron et al., 2001), it can degrade a large variety of algal polysaccharides. Sequencing of its genome revealed a number of adaptive traits for interactions with algae, such as, consumption of seaweed exudates, potential resistance to algal defense and a huge arsenal of CAZymes and sulfatases to degrade polysaccharides (Barbeyron et al., 2016b). Detailed biochemical studies have begun to unveil complex enzymatic systems for the degradation of agars (Hehemann et al., 2012) and carrageenans (Barbeyron et al., 1998; Rebuffet et al., 2010) from red algae and alginate (Thomas et al., 2012, 2013; Zhu et al., 2017) and laminarin (Labourel et al., 2014, 2015) from brown algae.

Here, we report the results of complete gene expression profiling for *Z. galactanivorans* Dsij^T^ during the utilization of algal polysaccharides. This approach allowed the prediction of operon-like genomic units throughout the chromosome. It further revealed polysaccharide-responsive regulons and new candidate genes for substrate recognition, transport, and degradation.

## Methods

### Substrates, bacterial strain, and culture conditions

The substrates tested for growth were glucose (Merck), alginate (Sigma), laminarin (Degussa), ι-carrageenan (Sanofi BioIndustries), κ-carrageenan (extracted from *Eucheuma cottonii*, gift from Goëmar), agar (Amresco), or porphyran (extracted from *Porphyra umbilicalis* as in Correc et al., 2011). ^1^H NMR analysis of the self-extracted porphyran and κ-carrageenan confirmed the purity and the motif composition of the polysaccharides (Correc et al., 2011; Préchoux et al., 2016). The type strain *Z. galactanivorans* Dsij^T^ (Barbeyron et al., 2001) was routinely grown from glycerol stocks in Zobell medium 2216E (Zobell, 1941) at 20°C. Cells were pelleted (5 min, 4,000 g) and resuspended in saline solution to remove traces of organic substrates. This suspension was inoculated (1/50 ratio) in triplicate flasks containing marine mineral medium (Thomas et al., 2011a) amended with 2 g.L^−1^ of one substrate as sole carbon source. Cultures were incubated at 20°C, 180 rpm. After 48 h, aliquots (5 ml) were centrifuged (10 min, 4,000 g) and cell pellets were resuspended in 200 μl Trizol reagent (Sigma), frozen in liquid nitrogen and stored at −80°C until RNA extraction.

### RNA preparation and cDNA synthesis

Samples were directly transferred from −80 to a 65°C water bath for 15 min to lyse cells and mixed with 50 μl chloroform for 5 min at room temperature. The aqueous phase was recovered after centrifugation for 15 min at 13,000 g, 4°C. Total RNA was purified using RNeasy Mini Kit (Qiagen) with on-column DNase I treatment following the manufacturer's instructions, and eluted in 15 μl RNase-free water. RNA concentration was quantified on a Nanodrop ND-1000 spectrophotometer (Nanodrop Technologies, Inc.). Concentrations and ratios A_260/280_ and A_260/230_ are given in Supplementary Table 1. RNA integrity was confirmed by 0.8% agarose gel electrophoresis. The absence of genomic DNA was confirmed by PCR on purified RNA. The cDNAs were synthesized from 10 μg total RNA using the Superscript Double Stranded cDNA kit (Invitrogen), purified by phenol:chloroform:isoamylic alcohol extraction (v/v, 25:24:1), recovered by ethanol precipitation and resuspended in 15 μl nuclease-free water. The cDNA quality was confirmed on a Bioanalyzer (Agilent) using the RNA 6000 Nano kit (Supplementary Figure 1).

### Microarray analysis

The transcriptomic analysis was performed by PartnerChip (Evry, France) on a custom microarray (4^*^72k, Roche Nimblegen). The whole genome of *Z. galactanivorans* Dsij^T^ (4,482 CDS + 2,595 intergenic regions) was covered by 72,000 oligonucleotides with a 60-bp average size. In total, the pangenomic microarray comprised 52,389 and 19,345 probes targeting genic and intergenic regions, respectively. It was impossible to design probes for 256 coding regions and 1,556 non-coding regions, either because sequences were too similar to ensure specificity or the target regions were too short. Overall, the microarray covered 95% of the genes of *Z. galactanivorans*, with 11.6 probes per gene on average. cDNA from each sample (1 μg) was labeled with Cyanine 3, hybridized on the microarray in a Nimblegen station and washed three times. Cyanine 3 fluorescence was scanned on a MS200 scanner (Roche Nimblegen). Raw intensities were obtained from the NimbleScan software (Nimblegen). The ANAIS web interface (Simon and Biot, 2010) was used to normalize data and detect significantly expressed genes, i.e., genes expressed more than random probes included on the chip (*p* < 0.01). Raw and normalized data were deposited on the NCBI GEO repository under accession GSE99940. Biological triplicate data obtained with each polysaccharide were compared to glucose condition using empirical Bayes statistics implemented in the *eBayes* function from the limma package v 3.26.9 in R 3.2.2 (R Core Team, 2013) and Bonferroni correction for multiple tests. Genes with an adjusted *p*-value FWER ≤ 0.05 were considered as differentially expressed. Non-metric multidimensional scaling (NMDS) was performed on a Morisita-Horn dissimilarity matrix obtained from normalized expression data with the vegan package (Oksanen et al., 2013). Sets of differentially expressed genes were compared using UpSetR (Lex et al., 2014).

### Detection of transcriptional units

In order to detect transcriptional units, a mathematical object called *Genomic & Transcriptomic Segment* (*GTSegment)* was defined. This object results from a combination of (i) a co-expression network built from the above-mentioned transcriptomic data and (ii) the *Z. galactanivorans* genome organization. First, a co-expression network was built as follows. Genes, and by extension intergenic regions, with the same transcriptomic behavior were identified by computing for each pair of genes *(g,g*′*)* the Spearman correlation *c*_(*g*,*g*′)_ and its related *p*-value *p*(*g*,*g*′). Two genes have a similar expression when *c*_(*g*,*g*′)_ ≥ 0.8 and *p*(*g*,*g*′) ≤ 10^−9^ (after Bonferroni correction for multiple tests). For the sake of validation, each significant correlation score was confirmed if identified by complementary *Mutual Information Coefficient* (MIC) (cv = 0.6) using MINE java programming (Reshef et al., 2011). We then built the co-expression network where nodes are the set of genes from *Z. galactanivorans* and edges connect two genes *g* and *g*′ when *g* and *g*′ have a similar expression. The co-expression network we obtained was composed of 3,912 nodes and 51,083 edges. For the sake of illustration, Supplementary Figure 2 represents the network via the use of a Hu algorithm, which groups highly interconnected nodes (Hu, 2005). In a second step, the *Z. galactanivorans* genome organization was modeled as a single circular sequence of genes, called *G*. The gene order in *G* is defined by the position of genes (and by extension intergenic regions) in the genome. A GTSegment *S* is then a sub-sequence of *G* where (i) *S* must contain at least 2 genes and at most 50 genes and (ii) a path in the co-expression network must exist between extremities of *S* only by passing through other genes from *S*. The quality of a GTSegment *S* is evaluated by its *density d*_*g*_*(S)*, which describes the proportion of genes in the GTSegment that behave similarly. Its formal definition is the following:

(1) $$d_{g}\left(S\right)=\frac{\left|R(S)\right|}{\left|S\right|}$$

where *R(S)* is the set of genes such as, each gene *g*″ in *R(S)* is a gene from *S* for which a path exists in the co-expression network from the extremities of *S* to *g*″ that only passes through other genes from *S*. GTSegments with density ≥0.6 are known to be good candidates for transcription units (Bordron et al., 2016).

As many GTSegments can be included within larger ones, we selected representative GTSegments, called *dominant GTSegments*. Given two GTSegments *A* and *B, B* dominates *A* if *R(A)* is a subset of *R(B)* (i.e., the part of the co-expression network involved in *A* is also involved in *B*), and *d*_*g*_*(A)* < *d*_*g*_*(B)* (i.e., the proportion of genes from the co-expression network involved in *B* is larger than the one involved in *A*). A dominant GTSegment is then a GTSegment that is not dominated by any other GTSegment.

The Python package developed to compute GTSegments is freely available at https://pypi.python.org/pypi/GTsegments

## Results and discussion

### Utilization of algal polysaccharides involves genome-wide transcriptomic shifts

To investigate transcriptomic changes linked to algal biomass utilization, *Z. galactanivorans* was grown in minimal medium with algal polysaccharides (alginate, laminarin, agar, porphyran, ι- or κ-carrageenan) or glucose used as a reference monosaccharide. These polysaccharides represent a large chemical diversity. Alginate is a polymer of 1,4-linked β-D-mannuronic acid and α-L-guluronic acid found in the cell wall of brown macroalgae. Laminarins are β-1,3 glucans with occasional β-1,6 branches used as carbon storage compounds in brown macroalgae and in microalgae such as, diatoms and oomycetes. Agar, porphyran, and carrageenan are sulfated galactans abundant in the cell wall of red macroalgae. They consist of a linear backbone of galactose residues linked by alternating β-1,4 and α-1,3 glycosidic bonds. The main difference lies in the configuration of the α-linked galactose units, which are in the L configuration in agar and porphyran and in the D configuration in carrageenans, and defines two groups of red macroalgae known as agarophytes and carrageenophytes. Agar and porphyran differ by the presence of a 3,6-anhydro bridge or the sulfurylation on the O6 in the α-L-galactose unit, respectively. ι- and κ-carrageenans both feature sulfate esters on the O4 of the β-linked D-galactose residues and 3,6-anhydro-bridges in the α-D-galactose residues, but differ in the presence of additional sulfate esters on the O2 of the α-D-galactose residues for ι-carrageenan. *Z. galactanivorans* grew best with glucose, laminarin, and porphyran, reaching cell density of 1 after 48 h when samples were collected for gene expression profiling (Supplementary Figure 3). In comparison, cell density only reached ca. 0.4 with alginate and agar, and ca. 0.2 with κ- and ι-carrageenans. This could reflect differences in substrate accessibility or the presence of refractory fractions in larger polysaccharides.

**Figure 1:**
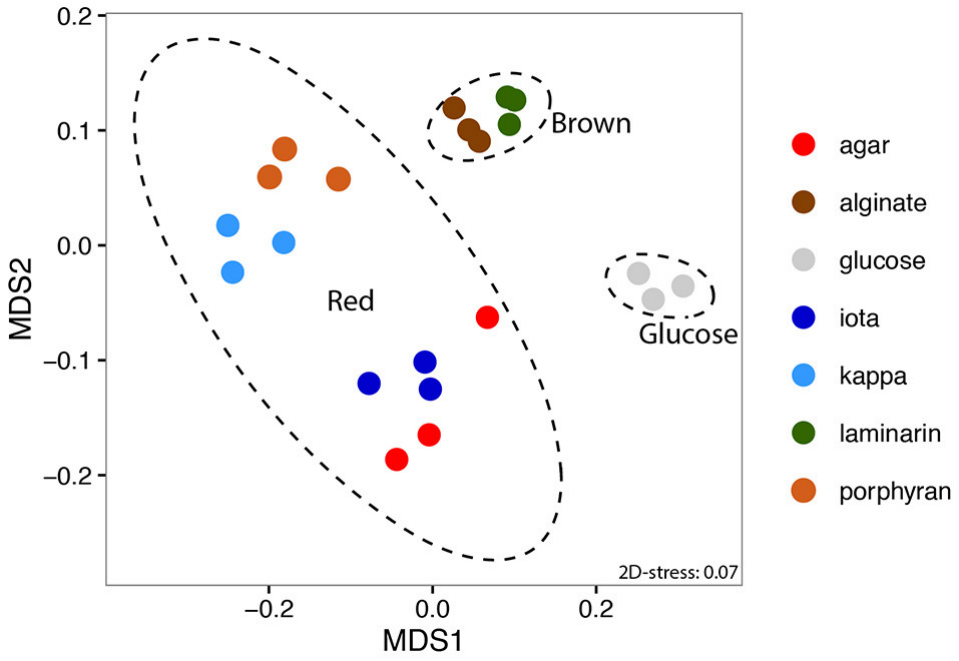
Non-metric Multi-Dimensional Scaling (NMDS) ordination of expression data based on Morisita-Horn dissimilarity. 95% confidence ellipses are depicted for the three groups of substrates (glucose, brown or red algal polysaccharides).

A total of 3,823 genes and 1,074 intergenic regions were detected as significantly expressed in at least one sample (*p* < 0.01). NMDS of expression data showed an overall consistency of the transcriptomic profiles obtained from triplicate cultures. Despite the difference in final cell density between samples, the procedure clearly separated three groups of profiles based on the origin of the substrates, namely (i) glucose, (ii) polysaccharides from brown algae, and (iii) polysaccharides from red algae (Figure 1). This shows that *Z. galactanivorans* transcriptomes share common features when cells use polysaccharides from the same algal phylum (*Phaeophyceae* vs. *Rhodophyceae*), The similarity in the transcriptomes of cells growing on red algal substrates might partly reflect the fact that agar, porphyran, and carrageenans are polymers containing D-galactose residues. Nonetheless, these polysaccharides still feature large structural differences. Notably, agars and porphyrans also contain L-galactose residues that are absent from carrageenans. The sulfation patterns of these polysaccharides are also very different. The similarity in these transcriptomes is thus unlikely to be explained only by the presence of D-galactose in red algal sulfated galactans. In the case of the brown algal polysaccharides, laminarin (glucose polymer) induced a transcriptomic profile that clusters tightly with that obtained on alginate (polymer of mannuronic and guluronic acid) and away from that obtained on glucose. Therefore, our results seem to indicate that common regulations occur in response to different substrates from the same algal phylum, triggering the activation of specific transcriptomic programs dedicated to the degradation of either brown or red algal biomass.

Differential expression analysis identified a set of 748 genes that were significantly up- or down-regulated (FWER ≤ 0.05, |log_2_FC| > 2) with at least one polysaccharide compared to glucose used as a control condition (Supplementary Table 2). The proportion of differentially expressed genes varied from 4.3 to 8.4% depending on the carbon source (Table 1). The number of differentially expressed genes was about twice greater with porphyran and κ-carrageenan than with alginate, laminarin, agar, and ι-carrageenan. This was mainly due to a higher number of down-regulated genes. These additional down-regulated genes (Supplementary Table 3) were involved in various cellular functions, including transcription (e.g., RNA polymerase), translation (e.g., ribosomal proteins), energy generation (e.g., ATP synthase), and glucose utilization (e.g., 2,3-bisphosphoglycerate-independent phosphoglycerate mutase, fructose bisphosphate aldolase, glucose-6-phosphate isomerase, glucose-6-phosphate-1-dehydrogenase). Unlike the four other polysaccharides, porphyran, and κ-carrageenan were not purchased commercially but rather purified directly from red macroalgae without acid or alkaline treatment. These extracted polysaccharides therefore likely better represent the complex structure of natural sulfated galactans found in red algal cell walls, such as, the presence of various substituents (sulfate, methyl, and pyruvate groups) that might be partially lost during the commercial preparation of polysaccharides. This could explain the larger effect on gene expression.

**Table 1:** Global regulation of transcriptome depending on carbon source.

**Carbon source**	**Up-regulated^a^**	**Down-regulated^a^**	**% regulated^b^**
Alginate	63 genes (2.0–4.3)	128 genes (2.0–5.1)	4.26%
Laminarin	102 genes (2.0–4.6)	99 genes (2.0–5.1)	4.48%
Agar	106 genes (2.0–5.6)	91 genes (2.0–5.1)	4.40%
Porphyran	47 genes (2.0–4.9)	308 genes (2.0–4.6)	7.92%
κ-Carrageenan	65 genes (2.0–5.3)	311 genes (2.0–5.1)	8.40%
ι-Carrageenan	91 genes (2.0–4.7)	111 genes (2.0–5.1)	4.51%

a *Differentially expressed genes compared to glucose condition (FWER < 0.05, |log_2_FC| > 2). The range of |log_2_FC| is given in brackets*.

b *Proportion of differentially expressed genes among all genes included on the chip*.

To validate the results, microarray data were compared with previously available RT-qPCR data obtained from independent experiments (Supplementary Figure 4). We considered the expression of 10 genes in the presence of laminarin, agar, porphyran, and alginate (Thomas et al., 2011a), as well as an additional set of 21 genes for alginate (Thomas et al., 2012). There was a strong positive correlation between log2 fold changes obtained previously by RT-qPCR and the microarray results [Pearson *r* = 0.823, *t*_(59)_ = 11.13, *p* < 0.001], confirming the robustness of the present data.

**Figure 2:**
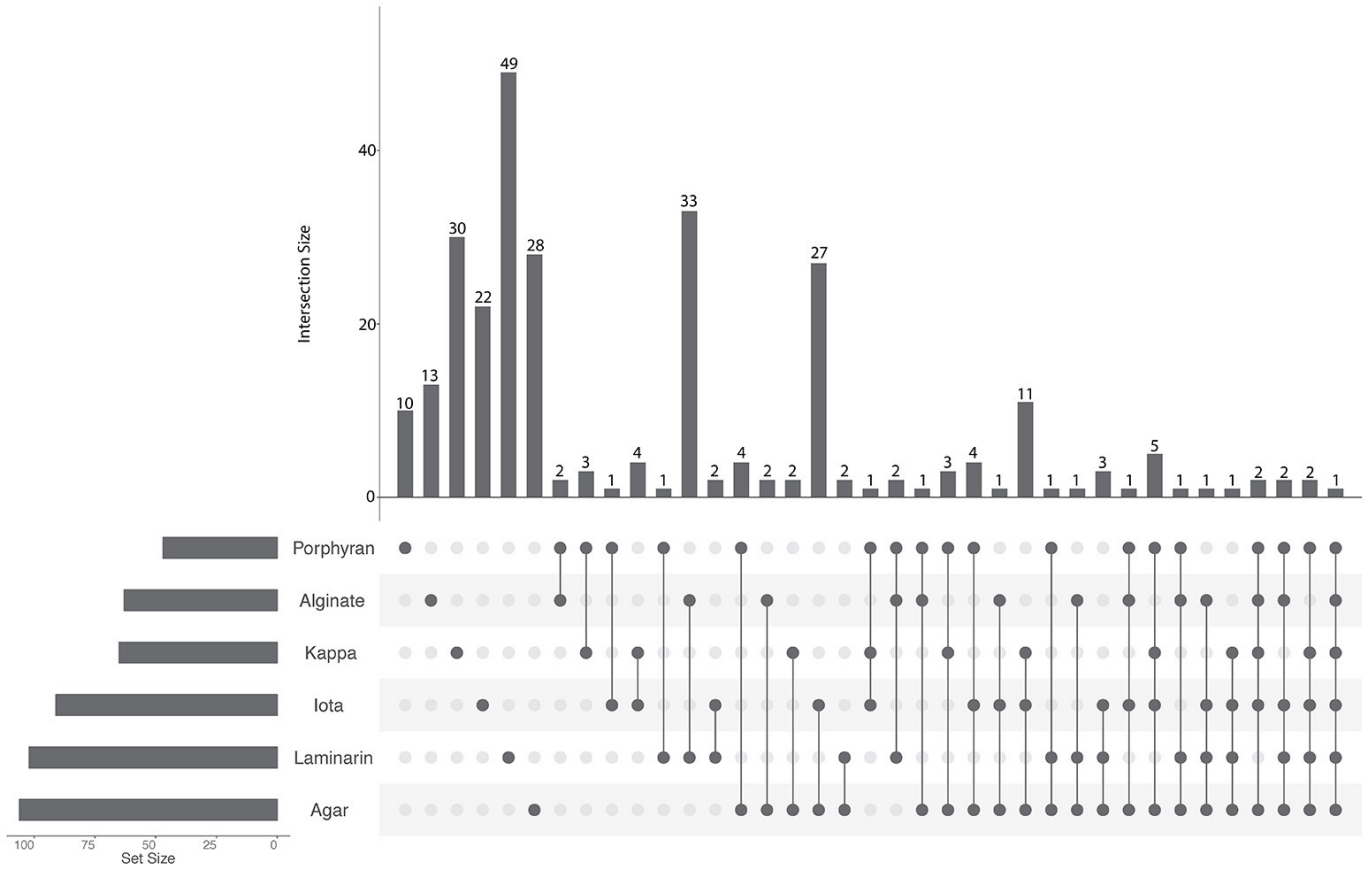
UpSet plot of intersections between sets of genes induced (FWER < 0.05, log_2_FC > 2) by each polysaccharide compared to the glucose condition. The bar chart on the left indicates the total number of up-regulated genes for each polysaccharide. The upper bar chart indicates the intersection size between sets of genes up-regulated with one or more polysaccharide(s). Dark connected dots on the bottom panel indicate which substrates are considered for each intersection.

To identify substrate-specific or shared regulations, we compared the sets of up-regulated genes obtained with each polysaccharide (Figure 2, Supplementary Table 4). Half of the up-regulated genes (152 out of 279) responded specifically to the presence of one polysaccharide. Among the remaining 127 genes that were up-regulated by at least two different substrates, the highest intersection size was found between the transcriptomes of cells growing with alginate or laminarin (33 genes, representing 52 and 32% of the total number of up-regulated genes with alginate or laminarin, respectively). Similarly, intersection sizes were high for genes responding to agar and ι-carrageenan (27 genes), agar, ι-carrageenan, and κ-carrageenan (11 genes), or the four red algal polysaccharides (5 genes). By contrast, the intersection size for groups up-regulated both with brown and red algal polysaccharides did not exceed three genes (Figure 2). This corroborates the hypothesis that polysaccharides from the same algal phylum trigger shared regulations.

### Expression profiling allows the detection of transcription units

**Figure 3:**
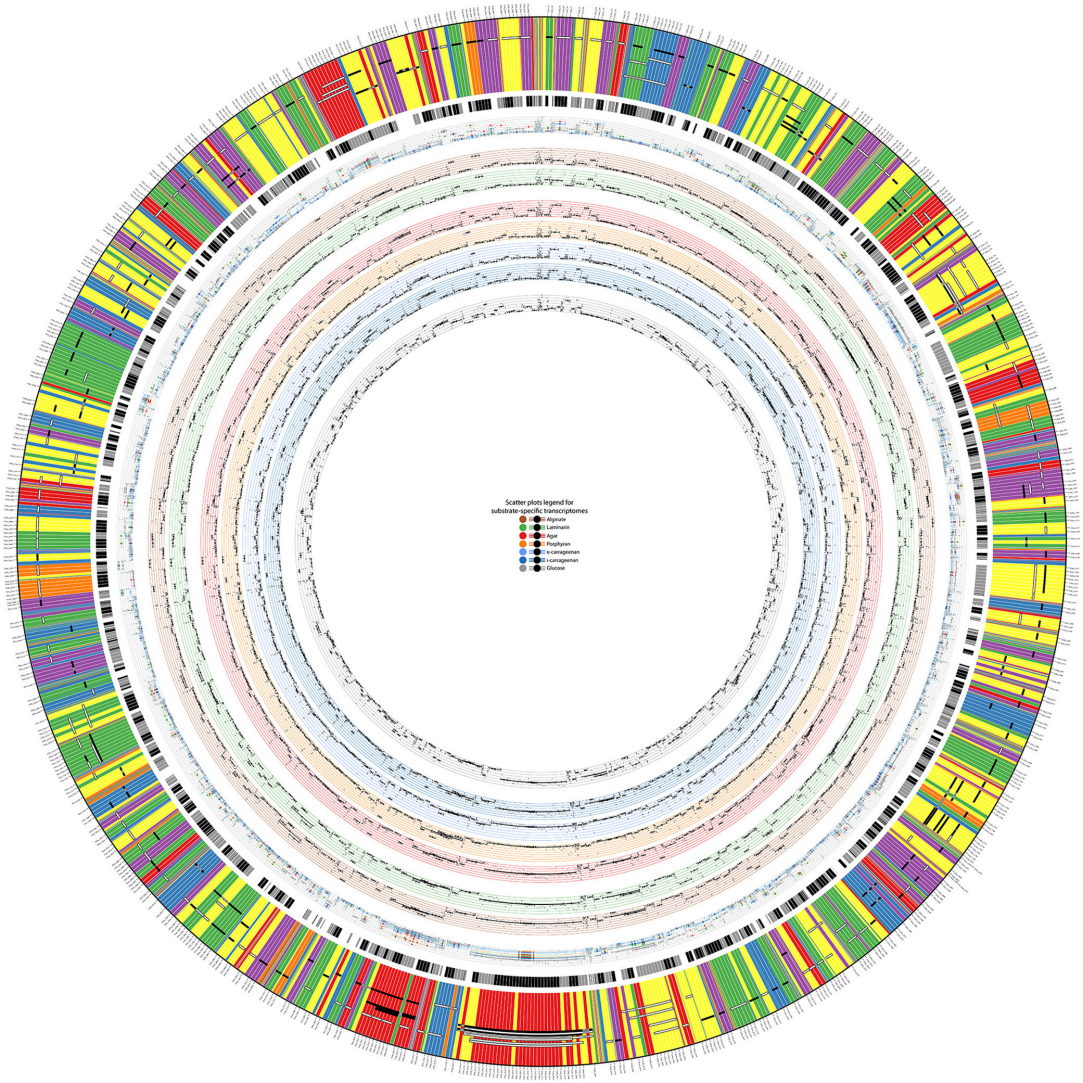
Display of detected transcription units (GTSegments) on *Z. galactanivorans* Dsij^T^ genome. The figure contains three main parts from the outer part to the inner part: the GTSegments, the predicted operons from DOOR2 database, and the expression values of genes and GTSegments in different growth conditions. The most outer band displays the sequence of genes existing in the chromosome of *Z. galactanivorans*. The color of each gene depicts its expression profile based on the co-expression network. Two genes with the same color have then the same expression profile. Black and white segments over the genes illustrate detected GTSegments. White segments are GTSegments similar to predicted operons in the DOOR2 database, whereas black segments are those that are not similar to predicted operons. Gray parts of a GTSegment indicate genes from the same locus that are omitted from the GTSegment. The second most outer band illustrates predicted operons from the DOOR2 database, where successive parts with the same color are genes from the same operon. Scatter plots on the inner rings depict expression values of genes and GTSegments in different growth conditions on a minimal medium with, from the most inner part to the outer part, glucose, ι-carrageenan, κ-carrageenan, porphyran, agar, laminarin, and alginate. The most outer scatter plot is the aggregation of all the previous plots, allowing thus to compare expression values at the same scale. In each plot, each small dot displays the expression value of a gene and each bold dot with whiskers displays the average expression value of a GTSegment.

The global transcription shift induced by the different tested conditions offered an opportunity to investigate transcription units in *Z. galactanivorans*. In prokaryotes, many genes are organized into operons, defined as multiple open reading frames transcribed from the same promoter to a single mRNA transcript. Operons allow co-regulation at the transcriptional level for genes that often act as part of the same pathway. These structures can be predicted based solely on sequence information, using distances between adjacent genes, presence of promoters and terminators, and conservation in other genomes (Ermolaeva et al., 2001). Such a sequence-based search applied to the *Z. galactanivorans* genome predicted 1,046 operons containing at least two genes, as reported on the Database of Prokaryotic Operons DOOR 2.0 (Mao et al., 2014). However, these predictions need to be validated experimentally. The microarray dataset was therefore used to infer transcription units (GTSegments), defined as groups of proximal genomic elements displaying a coordinated expression in the tested conditions. The computation of dominant GTSegments in *Z. galactanivorans* using a custom script produced 278 transcription units of more than two and up to 17 genes (Figure 3, also available as high-resolution file in Supplementary Figure 5; Supplementary Table 5). These transcription units represented 192 unique regions distributed over the entire genome. A large proportion of the 1,046 total operons predicted by DOOR was not detected from the co-expression network. This does not necessarily invalidate the DOOR predictions, but likely suggests that the set of tested growth conditions did not induce sufficient perturbations of the gene expression in all predicted operons to produce a detectable signal (Sabatti et al., 2002). Our experimental design, focused on the utilization of different algal carbon sources, is expected to better detect transcription units in the *Z. galactanivorans* genome for which the expression changes in the presence of polysaccharides.

A minority of 20% of the detected transcription units (55 GTSegments) featured genes transcribed in opposite directions (Supplementary Table 5), which necessarily belong to different operons. This co-expression of adjacent genes coded on opposite strands might arise from transcriptional coupling of closely spaced divergent promoters (Beck and Warren, 1988). By contrast, 80% of the detected transcription units (223 GTSegments) comprised genes transcribed all in the same direction and are therefore compatible with an operon structure. Of these, 136 transcription units were in agreement with the sequence-based DOOR predictions (Figure 3), providing empirical support for operon organization. A number of transcriptional units were represented by several GTSegments of different lengths, which could reflect the existence of secondary promoters within the operons, transcriptional attenuation, or differential degradation of mRNA. It is worth noting that this experimental approach without *a priori* succeeded in detecting transcription units known to be transcribed as genuine operons in other prokaryotic organisms. This includes operons involved in the biosynthesis of ribosomal proteins (Regions #25 and #93, Supplementary Table 5), tryptophan (Region #26), arginine (Region #47), an F1F0 ATPase complex (Region #65), and a PUL dedicated to starch degradation (Region #15). Furthermore, novel operon-like structures potentially involved in algal polysaccharide utilization were detected (detailed below).

### Polysaccharide-responsive regulons provide new candidate genes putatively involved in algal substrate recognition, transport, and degradation

**Figure 4:**
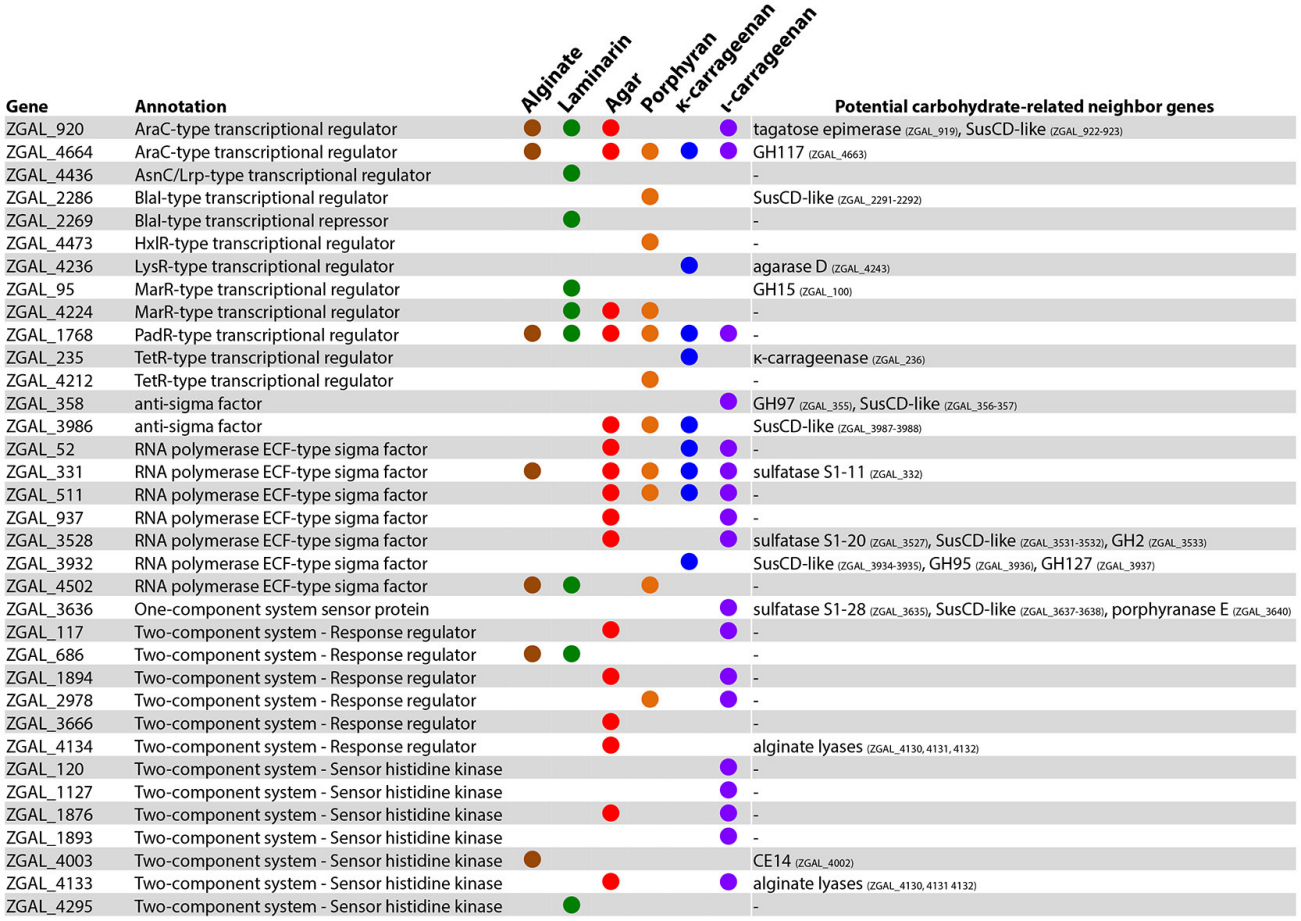
Genes involved in transcriptional regulation and induced with at least one polysaccharide (FWER < 0.05; log_2_FC > 2). A colored circle indicates that the gene was induced by a given substrate. For each regulator, genes potentially involved in carbohydrate catabolism found in the same genomic region are reported.

Among the 279 genes up-regulated with at least one of the polysaccharides (FWER ≤ 0.05, log_2_FC > 2), a large fraction (120 genes, i.e., 43%) encoded hypothetical proteins that lacked functional annotation. This number is significantly higher than what would be expected by chance, given that the microarray targets 1,429 genes encoding hypothetical proteins out of the 4,482 genes represented (Chi-squared test, χ^2^ = 15.9; *p* < 0.001). This enrichment of genes of unknown function within the polysaccharide-responsive regulons highlights the current lack of knowledge on catabolic pathways dedicated to marine polysaccharides, compared to glucose utilization for which the degradation machinery has been extensively studied. Furthermore, genes encoding proteins involved in transcriptional regulation were also significantly enriched among the up-regulated genes (Chi-squared test, χ^2^ = 15.0; *p* < 0.001). A total of 35 genes belonging to this category were up-regulated in at least one condition (Figure 4). This includes genes annotated as transcriptional regulators of the AraC, AsnC/Lrp, BlaI, HxlR, LysR, MarR, PadR, and TetR families, sigma and anti-sigma factors, or one- and two-component system proteins. Examination of the genomic region around each of these regulators revealed a number of potential carbohydrate-related genes, corroborating a role in the global cell response toward polysaccharide utilization (Figure 4). Half of the up-regulated regulators (17/35) responded specifically to one of the tested polysaccharides and likely participate to trigger the expression of substrate-specific pathways. On the other hand, some regulatory genes responded to different polysaccharides (Figure 4). These genes might be involved more generally in the adaptation of cells toward interaction with algal biomass. A striking example is the gene ZGAL_1768, encoding a putative PadR-type transcriptional regulator that was strongly up-regulated with all six polysaccharides tested (log_2_FC > 3), together with the downstream gene of unknown function ZGAL_1769 (Supplementary Figure 6). Both genes were found as part of a transcriptional unit in the coexpression network analysis (Supplementary Table 5). By contrast, the upstream gene *paaE* (ZGAL_1767), encoding a putative phenylacetic acid (PAA) degradation NADH oxidoreductase, was down-regulated with all six polysaccharides tested and negatively correlated with the expression of the ZGAL_1768 PadR-type regulator (Supplementary Figure 6). This is reminiscent of the role of some members of the large and diverse PadR-like family (PFAM accession PF03551) for the response to phenolic acids, including the prototypical PadR phenolic acid decarboxylation repressor that inhibits the expression of an inducible phenolic acid decarboxylase in gram-positive bacteria (Barthelmebs et al., 2000) and the VanR repressor controlling the vanillate utilization operon in *Corynebacterium glutamicum* (Heravi et al., 2015). This suggests that ZGAL_1768 could represent a new member of the PadR-like family repressing PAA degradation in *Z. galactanivorans*. PAA is known as a natural growth stimulator for green, red, and brown macroalgae (Fries and Iwasaki, 1976; Fries, 1977; Fries and Āberg, 1978). The up-regulation of ZGAL_1768 in response to algal polysaccharides might therefore participate to the adaptation of *Z. galactanivorans* as an alga-associated bacterium by promoting the repression of PAA degradation in the presence of macroalgae.

**Figure 5:**
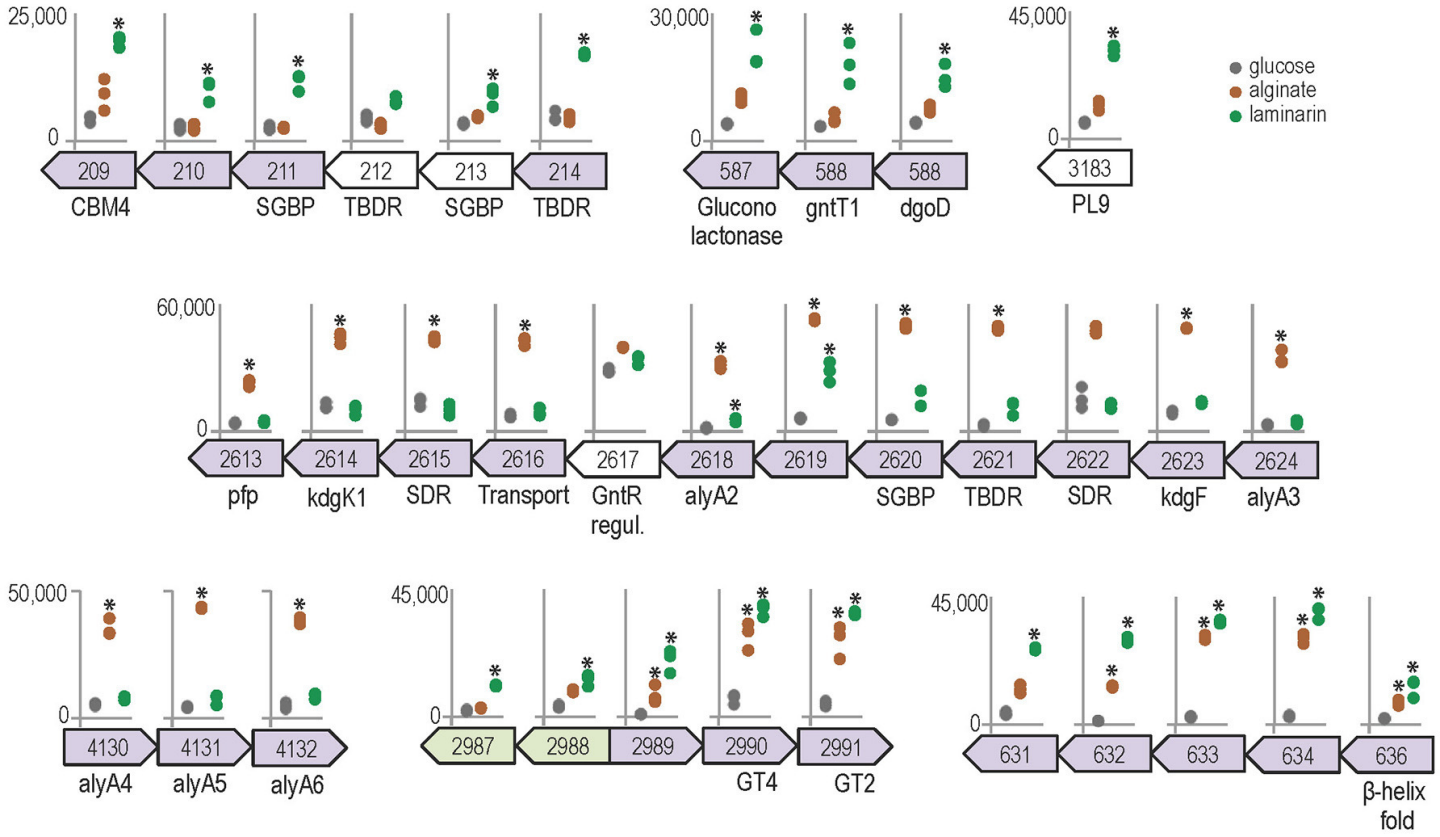
Genomic context and expression values of selected genes induced by alginate (brown) and/or laminarin (green) compared to glucose condition (gray). Asterisks denote significant up-regulation compared to glucose (FWER < 0.05). The y-axis scale reports normalized expression values and is conserved for each genomic region. Genes from the same genomic region and shaded with the same color were found to belong to the same transcription unit by the network approach.

Growth on polysaccharides also triggered the expression of genes and operon-like structures related to carbohydrate catabolism in *Z. galactanivorans*. Laminarin induced the expression of the transcriptional unit ZGAL_209 to ZGAL_214, which encodes two TonB-dependent receptors (TBDR) with their associated surface glycan-binding protein (SGBP) of the SusD-like family and a CBM4-containing hypothetical protein localized in the outer membrane (Figure 5). Tandems of TBDRs and SusD-like SGBPs are considered a hallmark of PULs in *Bacteroidetes* genomes (Grondin et al., 2017). To date, binding of CBM4 modules has been demonstrated with β-1,3-glucan, β-1,3-1,4-glucan, β-1,6-glucan, xylan, and amorphous cellulose (CAZY database, http://www.cazy.org/). Therefore, this operon-like structure could be involved in the binding of laminarin to the cell surface. The three genes ZGAL_587, ZGAL_588, and ZGAL_589 were also detected as a transcriptional unit and were significantly up-regulated when cells were grown on laminarin compared to glucose (Figure 5). The first of these genes encodes a periplasmic gluconolactonase, predicted to hydrolyze the laminarin degradation product gluconolactone to gluconate that in turn can be imported to the cytoplasm by the gluconate transporter GntT1 encoded by ZGAL_588. The gene ZGAL_589 is annotated as a cytoplasmic galactonate dehydratase *dgoD*. However, its induction by laminarin and its proximity with gluconate-related genes suggest that it rather converts gluconate to 2-dehydro-3-deoxy-D-gluconate. Laminarin further induced (log_2_FC = 2.4) the expression of ZGAL_3183, a member of the polysaccharide lyase family PL9, subfamily 4. This protein shares 32% identity with DssA from *Paenibacillus koleovorans*, which catalyzes the endolytic eliminative cleavage of (1
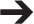
4)-α-galactosaminic bonds found in the sheath polysaccharide of *Sphaerotilus natans* (Kondo et al., 2011). Laminarin from the brown algae *Cystoseira barbata* (Stackhouse) C. Agardh 1820 and *C. crinita* Duby 1830 can contain a small percentage of N-acetylhexosamine-terminated chains (Chizhov et al., 1998). Induction of ZGAL_3183 by laminarin and homology with DssA might therefore point at new substrate specificity toward these non-canonical motifs.

The expression of genes from two detected transcriptional units was significantly increased in the presence of alginate, namely ZGAL_2613–2624 and ZGAL_4130–4132 (Figure 5). These regions were previously shown by RT-PCR to be transcribed as genuine operons, confirming the sensitivity of the co-expression network approach (Thomas et al., 2012). They encode the main components of the alginate-utilization system in *Z. galactanivorans*, notably biochemically validated alginate lyases and downstream processing enzymes, a transcriptional regulator and a TBDR/SGBP tandem (Thomas et al., 2012). These two regions are reminiscent of a set of genes that was recently found upregulated in the *Firmicutes B. weihaiensis* Alg07 when grown with kelp powder, with the important difference that it included ATP-binding cassette (ABC) importer genes instead of a TBDR/SGBP tandem (Zhu et al., 2016). ABC importers are mainly restricted to small solutes such as, free amino acids and sugars, whereas TBDR/SGBP systems can target high molecular weight compounds. Differences in substrate binding and import mechanisms might therefore point at contrasting ecological strategies for the degradation of brown algal biomass, with *Z. galactanivorans* performing initial attack on complex polysaccharides and *B. weihaiensis* preferentially using soluble degradation products. Such niche speciation between flavobacteria and other marine bacteria has already been proposed regarding the degradation of phytoplankton-derived organic matter (Teeling et al., 2012; Williams et al., 2013). In addition, the genes ZGAL_2990 and ZGAL_2991, encoding, respectively, glycosyltransferases of family GT4 and GT2, were up-regulated by both laminarin and alginate together with adjacent genes of unknown function (Figure 5). Similarly, the expression of the detected transcriptional unit ZGAL_631-636, encoding proteins of unknown function, was significantly enhanced with alginate or laminarin. The presence of Thrombospondin type 3 repeats (IPR028974, four in ZGAL_631, one in ZGAL_633) and Calx-beta domains (PF03160, one in ZGAL_631, two in ZGAL_636) suggests a role in calcium binding. Furthermore, ZGAL_636 contains a domain of the CUB family, some members of which are believed to function in protein/carbohydrate interactions (Töpfer-Petersen et al., 2008), and a C-terminal β-helix fold domain resembling that of PL and GH28 families. Therefore, ZGAL_636 might represent a new CAZyme family of unknown specificity.

**Figure 6:**
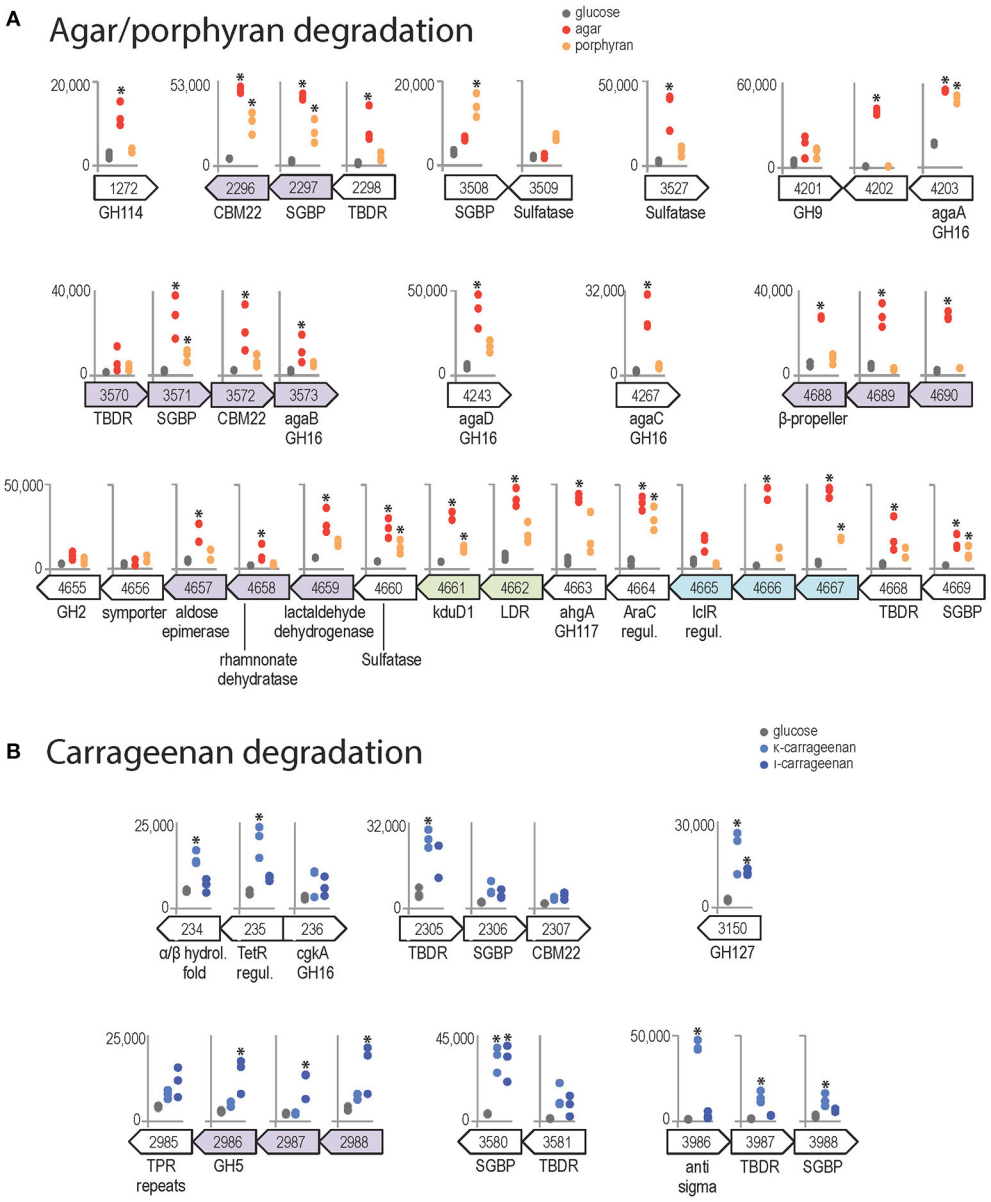
Genomic context and expression values of selected genes induced by red algal polysaccharides (FWER < 0.05). Asterisks denote significant up-regulation compared to glucose (FWER < 0.05). The y-axis scale reports normalized expression values and is conserved for each genomic region. Genes from the same genomic region and shaded with the same color were found to belong to the same transcription unit by the network approach. **(A)** Genes induced by agar (red) and/or porphyran (orange) compared to glucose condition (gray). **(B)** Genes induced by κ-carrageenan (light blue) and/or ι-carrageenan (dark blue) compared to glucose condition.

Agar induced the expression of the four β-agarases AgaA-D (Figure 6A), which have complementary roles in the complex *Z. galactanivorans* agarolytic system. AgaA and AgaD are secreted enzymes, thought to be specialized in the initial attack of solid-phase agars (Jam et al., 2005; Hehemann et al., 2012). AgaA expression was also induced by porphyran, corroborating its tolerance for sulfated moieties as shown previously (Hehemann et al., 2012). A gene of unknown function ZGAL_4202 localized next to *agaA* was highly expressed with agar and thus might play a yet unclear role for agar catabolism. The soluble oligosaccharides produced by AgaA and/or AgaD can be further processed by the outer membrane-bound AgaB (Jam et al., 2005). *agaB* is part of a detected transcriptional unit with genes encoding a TBDR, a SusD-like SGBP and a CBM22-containing lipoprotein. The expression of the latter two genes was induced with agar (Figure 6A). The paralogous gene cluster encompassing ZGAL_2296 (42% similarity with ZGAL_3572), ZGAL_2297 (48% similarity with ZGAL_3571), and ZGAL_2298 (62% similarity with ZGAL_3570) was also strongly expressed with agar. This organization is reminiscent of a PUL organization, suggesting a role for the binding and import of agaro-oligosaccharides into the periplasm. Furthermore, a large cluster ZGAL_4657 to ZGAL_4669 was significantly induced in the presence of agar and to a lesser extent of porphyran. It contains the *ahgA* gene coding for an exolytic 3,6 anhydro-α-L-galactosidase of the GH117 family, which releases 3,6-anhydro-L-galactose (L-AnG) from agaro-oligosaccharides (Rebuffet et al., 2011). Other genes from the cluster show strong sequence identities (Supplementary Table 6) with genes involved in L-AnG catabolism in *Pseudoalteromonas atlantica* T6c and *Vibrio natriegens* EJY3 (Lee S. B. et al., 2014; Yun et al., 2015). This cluster also encodes a TBDR/SGBP tandem, two transcriptional regulators and a sulfatase of the S1-19 family (Barbeyron et al., 2016a). Although not induced by agars in our experiments, the gene ZGAL_4655 next to the L-AnG catabolism cluster has 48% sequence identity with the β-galactosidase VEJY3_09170 from *V. natriegens* EJY3 (Supplementary Table 6), which releases galactose residues from the non-reducing end of agaro-oligosaccharides (Lee C. H. et al., 2014). Therefore, we discovered a substrate-inducible PUL for agaro-oligosaccharides in *Z. galactanivorans* that complements the action of the above-mentioned agarases. Two additional sulfatases are good candidates to desulfate agars, namely ZGAL_3527 (S1-20 family, expression induced 12-fold with agar compared to glucose) and ZGAL_3509 (S1-16 family), which is located next to a predicted SGBP induced with porphyran (Figure 6A). Finally, results point at agar-responsive genes for which a link with agar catabolism is still unclear, including ZGAL_1272 and the detected transcriptional unit ZGAL_4688 to ZGAL_4690. ZGAL_1272 encodes a putative glycoside hydrolase from the GH114 family, in which only one member was characterized as an endo-α-1,4-polygalactosaminidase (Tamura et al., 1995). However, the two sequences only share 25% identity (data not shown), suggesting different substrate specificities. ZGAL_4688 encodes a protein that likely adopts a beta-propeller fold (InterProScan domain IPR011042), which might be reminiscent of the structure found in several known GH families.

Both κ- and ι-carrageenan induced the expression of ZGAL_3150, annotated as a glycoside hydrolase of family GH127 (Figure 6B). The only characterized activities in the GH127 family are the hydrolysis of β-L-arabinofuranose from plant extensins (Fujita et al., 2014) and of 3-C-Carboxy-5-deoxy-L-xylofuranose from pectin (Ndeh et al., 2017). These sugars have not been found in red algae, suggesting a new substrate specifity on carrageenan motifs for ZGAL_3150. Both carrageenans also strongly induced the expression of a SusD-like SGBP (ZGAL_3580, log_2_FC > 3) adjacent to a TBDR gene, suggesting a role in binding of carrageenan to the cell surface. κ-carrageenan, but not ι-carrageenan, further induced the expression of another TBDR/SGBP pair (ZGAL_3987/ZGAL_3988) and one TBDR (ZG_2305), which might have concerted roles for substrate binding. In addition, the two adjacent genes ZGAL_234 and ZGAL_235 were up-regulated during growth with κ-carrageenan (Figure 6B). These two genes are adjacent to ZGAL_236, which encodes the characterized κ-carrageenase CgkA (Barbeyron et al., 1998). ZGAL_235 encodes a putative transcriptional regulator of the TetR family. ZGAL_234 encodes a protein with an alpha/beta hydrolase-fold containing an N-terminal haloalkane dehalogenase domain that shares 40% sequence identity with that of the characterized enzyme DbjA from *Bradyrhizobium japonicum* 3I1B110 (Sato et al., 2005). The up-regulation of ZGAL_234 by carrageenan might therefore help the bacteria when degrading cell walls, to cope with halogenated compounds that are released by red algae under stress conditions (Cosse et al., 2007). Finally, the expression of the locus ZGAL_2985–2988 was induced by ι-carrageenan, but not by κ-carrageenan (Figure 6B). It encodes three proteins of unknown function and a glycoside hydrolase of family 5, subfamily 42 (ZGAL_2986). GH5 is one of the largest of all CAZy families, encompassing a variety of activities. To date, GH5_42 counts 54 members in the CAZy database (http://www.cazy.org/GH5_42_all.html) but their substrate specificity remains unknown since all of them lack experimental characterization (Aspeborg et al., 2012). Our results could therefore guide future activity tests on ι-carrageenan related substrates.

In summary, this study provided an unprecedented view of genome-wide expression changes in an alga-associated flavobacterium during polysaccharide degradation. This integrative approach succeeded in detecting substrate-induced regulation of the expression of several characterized enzymes acting on algal polymers, such as, a 3,6-anhydro-L-galactosidase, agarases, and alginate lyases. It also revealed a set of candidate genes potentially representing new substrate specificities, and will guide future biochemical characterization attempts. In addition, transcriptomes shared common features when growing on chemically divergent polysaccharides from the same algal phylum. Together with the induction of numerous transcription factors, this hints at complex regulation events that fine-tune the cell behavior during interactions with algal biomass in the marine environment.

## Author contributions

FT and GM conceived and designed the experiments. FT carried out the experiments. PB and DE developed the script to detect transcription units. FT, PB, and DE analyzed the results. All authors discussed the results and assisted in writing the manuscript.

### Conflict of interest statement

The authors declare that the research was conducted in the absence of any commercial or financial relationships that could be construed as a potential conflict of interest.
