# Supplementary material for: Gene Expression Analysis of Zobellia galactanivorans during the Degradation of Algal Polysaccharides Reveals both Substrate-Specific and Shared Transcriptome-Wide Responses
Source: Front Microbiol. 2017 Sep 21;8:1808. doi: 10.3389/fmicb.2017.01808 (PMC5613140; doi:10.3389/fmicb.2017.01808)
Supplement: Supplementary file 1 [file Table1.DOCX]

**Supplementary Table 1: Quantification of RNA samples**

| **Sample** | **[RNA] µg.µl^-1^** | **A_260/280_** | **A_260/230_** |
| --- | --- | --- | --- |
| Glucose 1 | 2.373 | 2.06 | 2.38 |
| Glucose 2 | 3.593 | 2.13 | 1.60 |
| Glucose 3 | 1.510 | 2.09 | 1.30 |
| Alginate 1 | 3.795 | 2.15 | 2.53 |
| Alginate 2 | 3.365 | 2.17 | 2.27 |
| Alginate 3 | 4.152 | 2.14 | 2.53 |
| Laminarin 1 | 2.544 | 2.13 | 2.33 |
| Laminarin 2 | 3.878 | 2.16 | 2.41 |
| Laminarin 3 | 5.098 | 2.15 | 2.36 |
| Porphyran 1 | 4.269 | 2.14 | 2.29 |
| Porphyran 2 | 2.728 | 2.14 | 2.56 |
| Porphyran 3 | 3.518 | 2.12 | 2.55 |
| Agar 1 | 1.198 | 2.16 | 2.14 |
| Agar 2 | 1.030 | 2.08 | 2.39 |
| Agar 3 | 0.909 | 2.22 | 2.09 |
| κ-carrageenan 1 | 1.689 | 2.09 | 2.32 |
| κ-carrageenan 2 | 1.635 | 2.08 | 2.37 |
| κ-carrageenan 3 | 1.704 | 2.09 | 2.38 |
| ι-carrageenan 1 | 9.455 | 2.14 | 2.43 |
| ι-carrageenan 2 | 6.675 | 2.13 | 2.30 |
| ι-carrageenan 3 | 6.397 | 2.11 | 2.37 |
