# Supplementary figures and images for: Gene Expression Analysis of Zobellia galactanivorans during the Degradation of Algal Polysaccharides Reveals both Substrate-Specific and Shared Transcriptome-Wide Responses
Source: Front Microbiol. 2017 Sep 21;8:1808. doi: 10.3389/fmicb.2017.01808 (PMC5613140; doi:10.3389/fmicb.2017.01808)

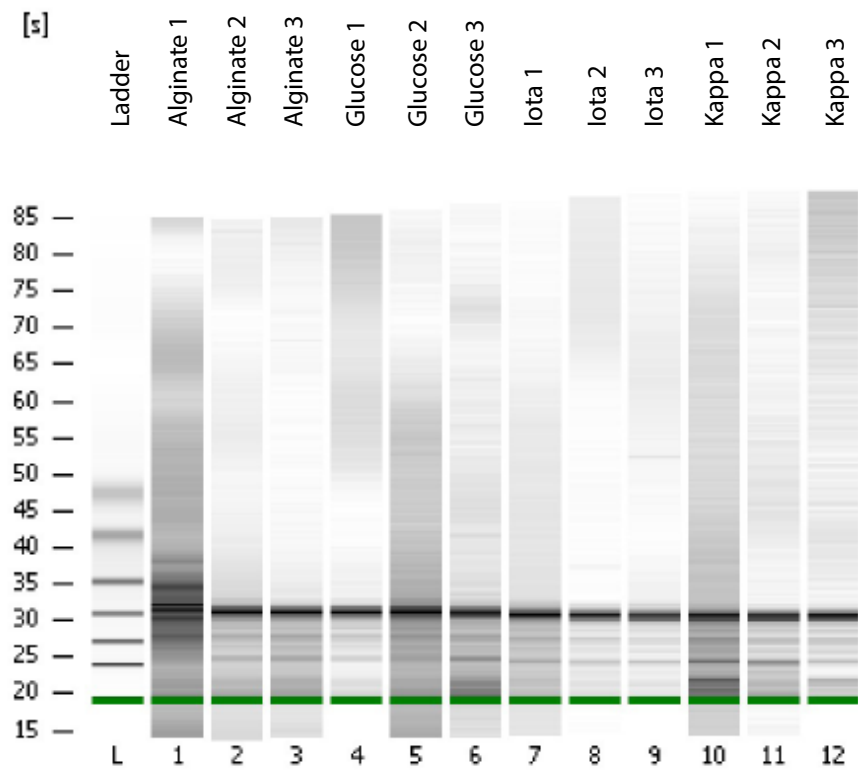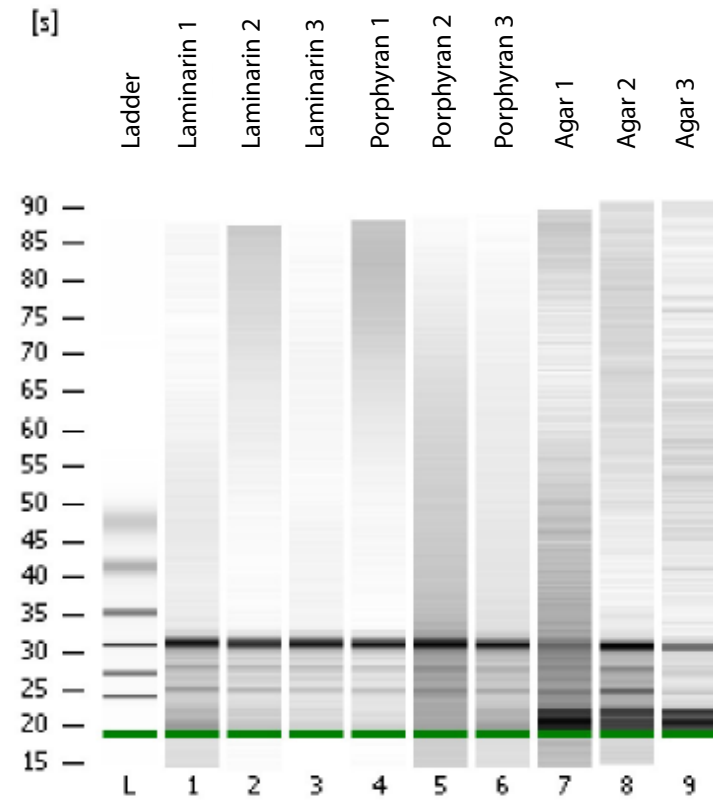

Supplement: Supplementary file 7 [file Image1.PDF]

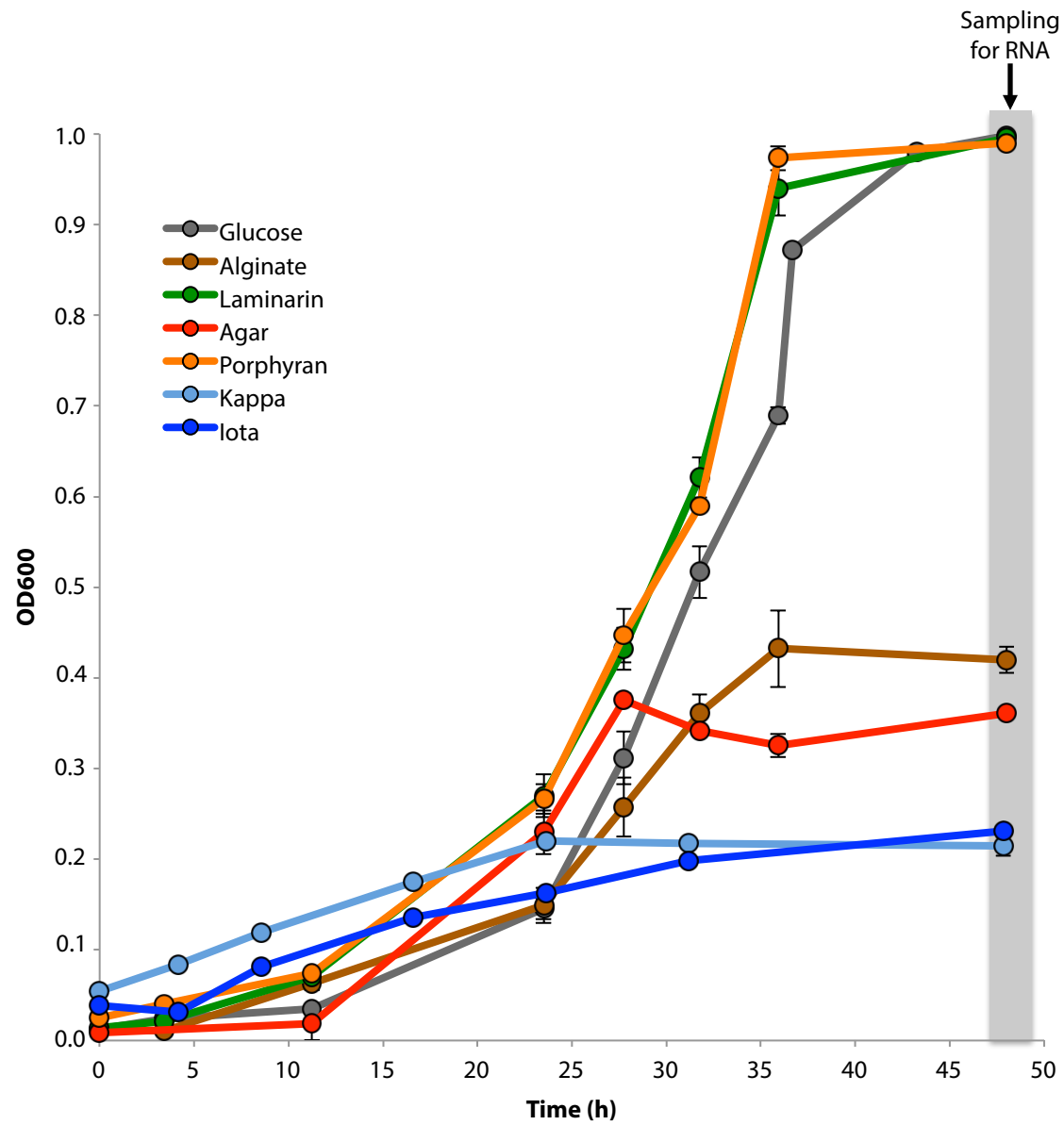

Supplement: Supplementary file 9 [file Image3.PDF]

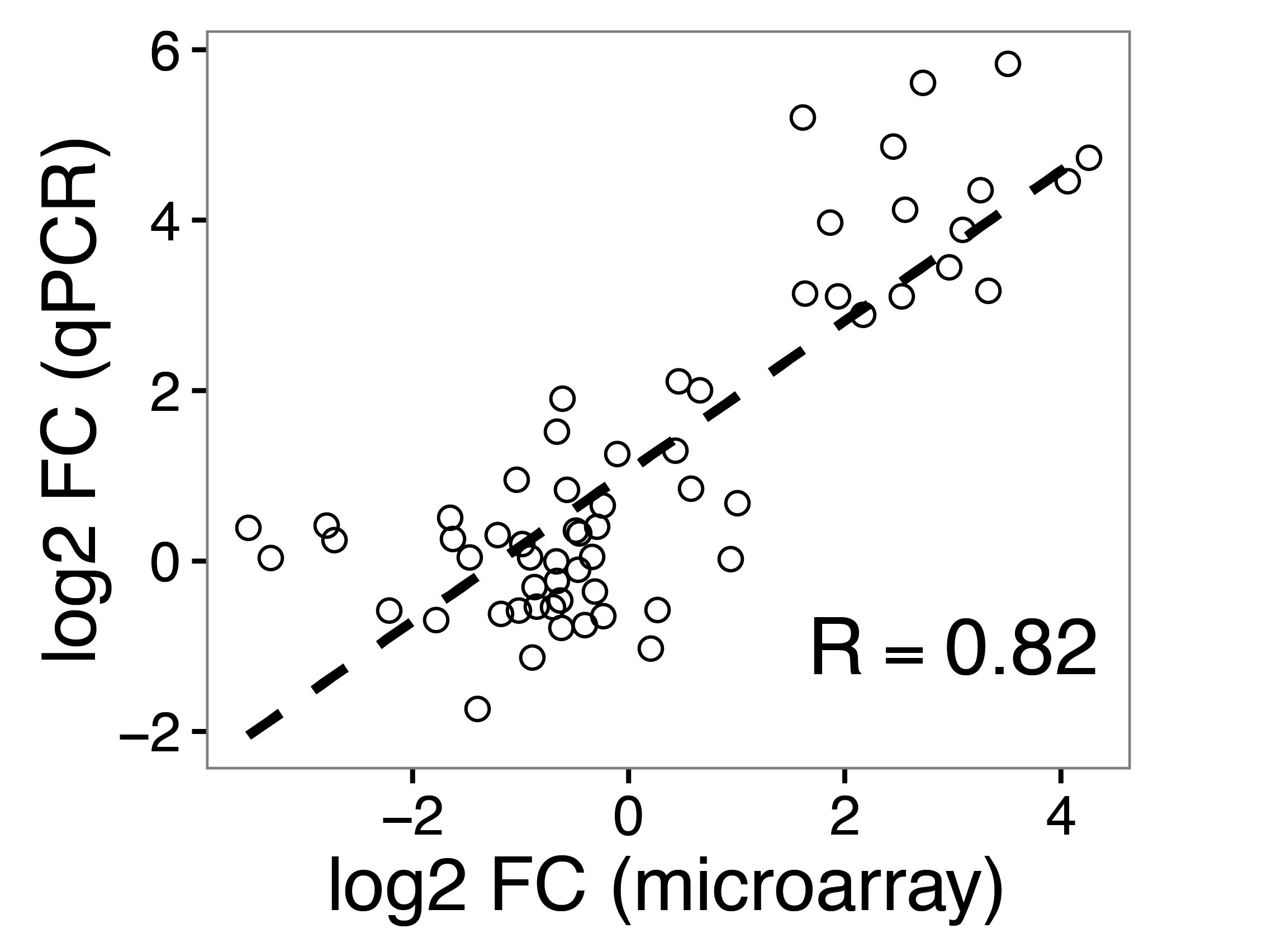

Supplement: Supplementary file 10 [file Image4.PNG]

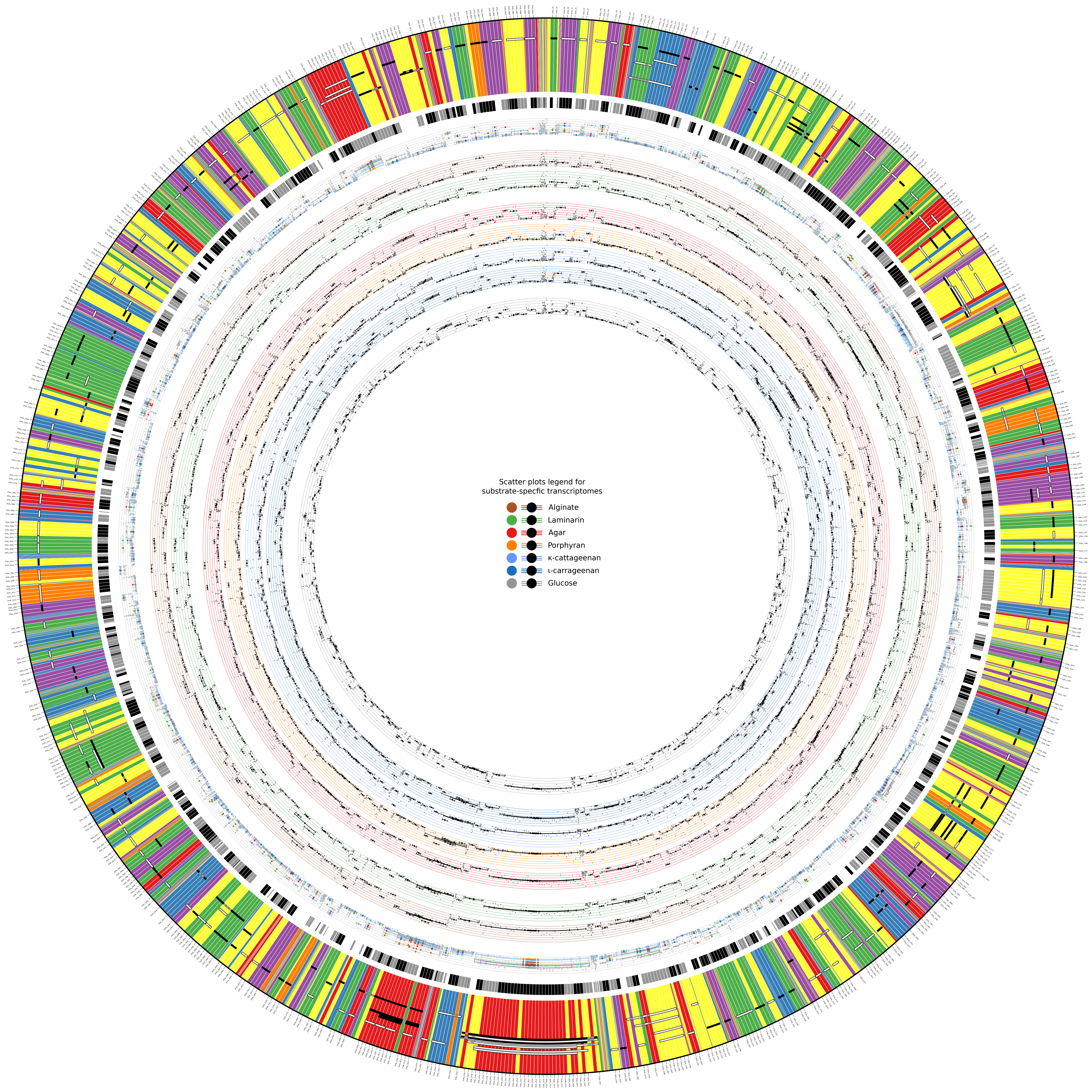

Supplement: Supplementary file 11 [file Image5.pdf]

**A.**

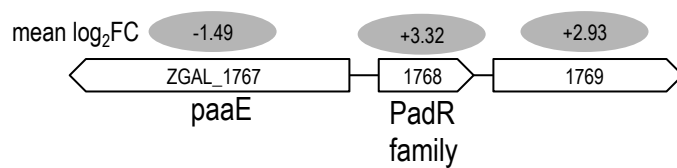

**B.**

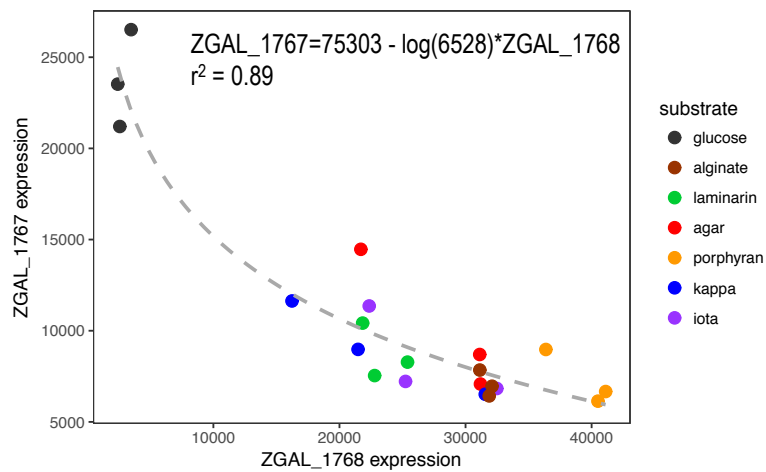

Supplement: Supplementary file 12 [file Image6.PDF]
