# Supplementary material for: Gene Expression Analysis of Zobellia galactanivorans during the Degradation of Algal Polysaccharides Reveals both Substrate-Specific and Shared Transcriptome-Wide Responses
Source: Front Microbiol. 2017 Sep 21;8:1808. doi: 10.3389/fmicb.2017.01808 (PMC5613140; doi:10.3389/fmicb.2017.01808)
Supplement: Supplementary file 8 [file Image2.PDF]

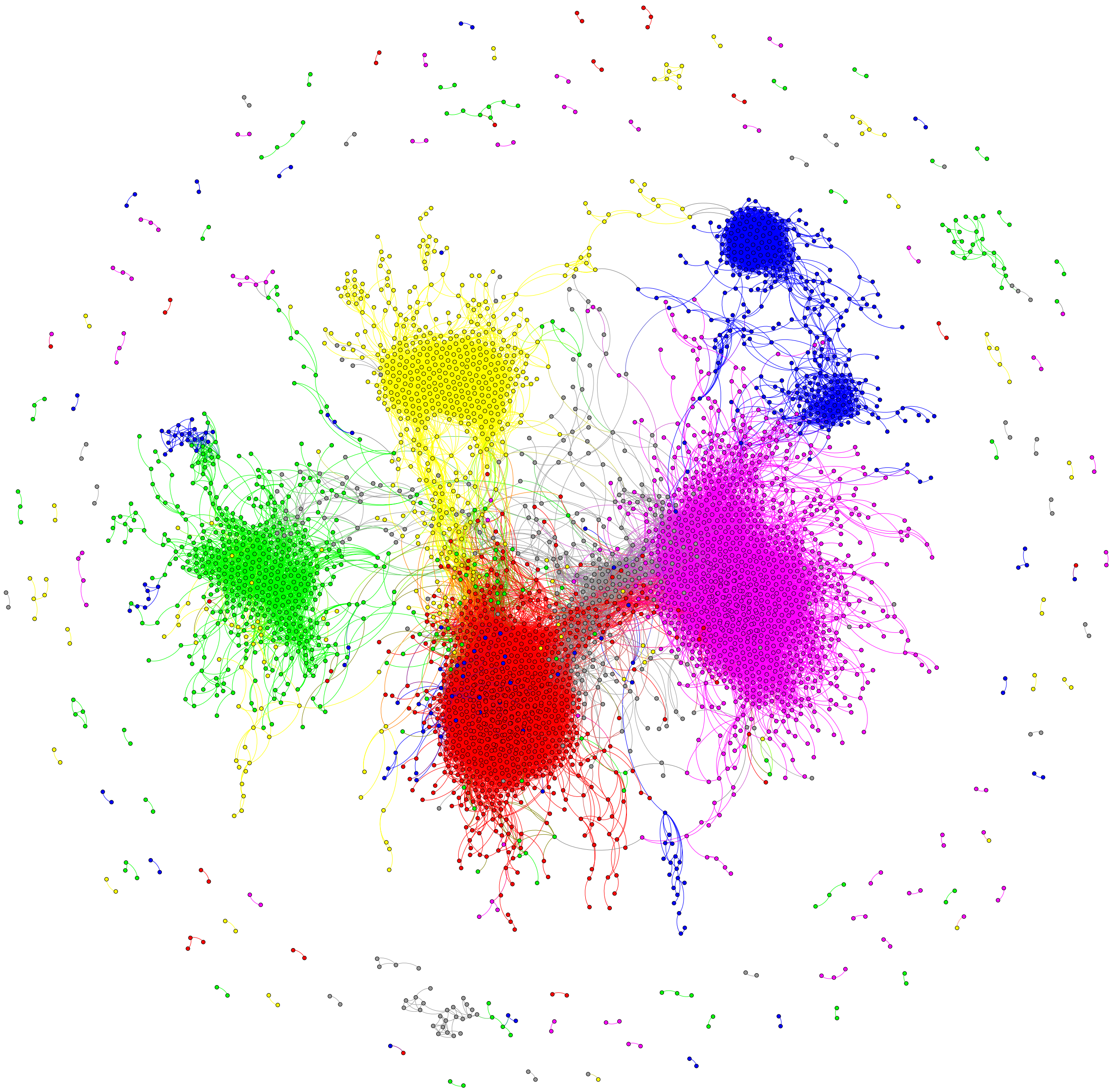

Supplementary Figure 2: *Zobellia galactanivorans* Dsij^T co-expression network colored by gene expression profiles. Each gene (i.e., node) is linked to another when their expressions are correlated (i.e., correlation higher than 0.8 and p-value below 10^{-9}, Bonferroni correction for multiple test). The Hu spatialization algorithm, used to display the co-expression network, spatially groups nodes that are highly interconnected to emphasize sets of co-expressed genes. Nodes acting as hubs point at genes of major interest because of their putative central role in co-expression. In parallel, a dual hierarchical analysis based on Spearman correlation was performed to assign genes to one of five categories of expression profile. Each category is depicted using a different color. The congruence of node spatialization and categories of expression profiles (i.e., non random distribution of colored nodes) validates the relevance of the gene clusters and the need for further investigations via the integration of genomic structure to point genes of particular interest.
