# Supplementary material for: Gene Expression Analysis of Zobellia galactanivorans during the Degradation of Algal Polysaccharides Reveals both Substrate-Specific and Shared Transcriptome-Wide Responses
Source: Front Microbiol. 2017 Sep 21;8:1808. doi: 10.3389/fmicb.2017.01808 (PMC5613140; doi:10.3389/fmicb.2017.01808)
Supplement: Supplementary file 13 [file Presentation1.PDF]

Supplementary Material

Gene expression analysis of *Zobellia galactanivorans* during the degradation of algal polysaccharides reveals both substrate-specific and shared transcriptome-wide responses

François Thomas[1,*] , Philippe Bordron[2], Damien Eveillard[3] & Gurvan Michel[1,*]

[1] Sorbonne Universités, UPMC Univ Paris 06, CNRS, UMR 8227, Integrative Biology of Marine Models, Station Biologique de Roscoff, Roscoff, France

[2] Sorbonne Universités, UPMC Univ Paris 06, CNRS, FR2424, Analysis and Bioinformatics for Marine Science, Station Biologique de Roscoff, Roscoff, France

[3] Université de Nantes, Laboratoire des Sciences du Numérique de Nantes, CNRS, ECN, IMTA, Nantes, France

[*] Correspondence: francois.thomas@sb-roscoff.fr; gurvan.michel@sb-roscoff.fr

Supplementary Figures and Tables Legends

Supplementary Table 1: Quantification of RNA samples

Supplementary Table 2: (excel file) Normalized expression data measured for each gene in *Z. galactanivorans* grown with glucose, alginate, laminarin, agar, porphyran, κ- or ι-carrageenan as sole carbon source. The log2-fold change compared to glucose is reported, together with the p-value of eBayes test for significance after Bonferroni correction for multiple testing.

Supplementary Table 3: Subset of Supplementary Table 2 showing genes that were significantly down-regulated (FWER≤0.05, $\log_2$FC<-2) with porphyran and κ-carrageenan but not with alginate, laminarin, agar or ι-carrageenan, compared to glucose. The log2-fold change compared to glucose is reported, together with the p-value of eBayes test for significance after Bonferroni correction for multiple testing.

Supplementary Table 4: Intersection sets of genes up-regulated with one or several polysaccharide(s) compared to glucose condition. This data was represented graphically in Figure 2.

Supplementary Table 5: (excel file) List of GTSegments detected in *Z. galactanivorans* based on the present expression data. Segments corresponding to similar loci have the same Region #.

Supplementary Table 6: (excel file) Genes in the agar-induced agarolytic cluster from *Z. galactanivorans* and their homologs in *Pseudoalteromonas atlantica* T6C and *Vibrio natriegens* EJY3. The encoded proteins can release and process 3,6-anhydro-L-galactose (L-AnG). This includes a 3,6 anhydro-α-L-galactosidase (ZGAL_4663) that releases L-AnG, and L-AnG dehydrogenase (ZGAL_4659) and 3,6-anhydro-L-galactonate cycloisomerase (ZGAL_4658) that sequentially convert L-AnG to 2-keto-3-deoxy-L-galactonate (L-KDGal). L-KDGal can further be processed into 2-keto-3-deoxy-D-gluconate (KDG) by a 2-keto-3-deoxy-L-galactonate 5-dehydrogenase (ZGAL_4662) and a 2,5-diketo-3-deoxy-L-galactonate 5-reductase (ZGAL_4661).

Supplementary Figure 1: cDNA profiles obtained on Agilent Bioanalyzer before labeling and hybridization.

Supplementary Figure 2: *Zobellia galactanivorans* Dsij^T co-expression network colored by gene expression profiles. Each gene (i.e., node) is linked to another when their expressions are correlated (i.e., correlation higher than 0.8 and p-value below 10^{-9}, Bonferroni correction for multiple test). The Hu spatialization algorithm, used to display the co-expression network, spatially groups nodes that are highly interconnected to emphasize sets of co-expressed genes. Nodes acting as hubs point at genes of major interest because of their putative central role in co-expression. In parallel, a dual hierarchical analysis based on Spearman correlation was performed to assign genes to one of five categories of expression profile. Each category is depicted using a different color. The congruence of node spatialization and categories of expression profiles (i.e., non random distribution of colored nodes) validates the relevance of the gene clusters and the need for further investigations via the integration of genomic structure to point genes of particular interest.

Supplementary Figure 3: Growth curves of *Zobellia galactanivorans* in minimum medium supplemented with one substrate as sole carbon source. Values are mean ± s.d. (n=3).

Supplementary Figure 4: Comparison of microarray and RT-qPCR data. Log2 fold changes (FC) against the glucose condition were calculated for 10 genes in the presence of laminarin, agar, porphyran and alginate, and an additional set of 21 genes for alginate (total: 61 comparisons). A linear regression was fitted (dotted line), and Pearson correlation indicated a strong correlation ($r = 0.823$, $t_{59} = 11.13$, $p<0.001$).

Supplementary Figure 5: Zoomable high-resolution version of Figure 3.

Supplementary Figure 6: A. Genomic context of the PadR-like transcriptional regulator (ZGAL_1768). For each gene, the mean log2FC with all six polysaccharides compared to glucose is given in gray ellipses. **B**. Scatterplot of the expression values obtained for ZGAL_1767 and ZGAL_1768 in each of the seven culture conditions. The equation and correlation coefficient of a log-regression model fitted to the data (dotted line) are reported.
